# DAXX mediates high phosphate-induced endothelial cell apoptosis in vitro through activating ERK signaling

**DOI:** 10.7717/peerj.9203

**Published:** 2020-06-19

**Authors:** Shu Wang, Mingyu Wu, Ling Qin, Yaxiang Song, Ai Peng

**Affiliations:** Nephrology and Rheumatology, Shanghai Tenth People’s Hospital, School of Medicine, Tongji University, Shanghai, China

**Keywords:** Hyperphosphatemia, Chronic kidney diseases, HUVECs, DAXX, ERK

## Abstract

**Backgroud and Purpose:**

Hyperphosphatemia, which is a high inorganic phosphate (Pi) level in the serum, promotes endothelial cells dysfunction and is associated with cardiovascular diseases in patients with chronic kidney diseases (CKD). However, the underlying mechanism of high Pi-induced endothelia cell apoptosis remains unclear.

**Methods:**

Human umbilical vein endothelial cells (HUVECs) were treated with normal Pi (1.0 mM) and high Pi (3.0 mM), and then cell apoptosis, abnormal gene expression and potential signaling pathway involvement in simulated hyperphosphatemia were examined using flow cytometry, quantitative PCR (qPCR) and western blot analysis. A two-step 5/6 nephrectomy was carried out to induce CKD and biochemical measurements were taken.

**Results:**

The rat model of CKD revealed that hyperphosphatemia is correlated with an increased death-domain associated protein (DAXX) expression in endothelial cells. In vitro, high Pi increased the mRNA and protein expression level of DAXX in HUVECs, effects that were reversed by additional phosphonoformic acid treatment. Functionally, high Pi resulted in a significantly increased apoptosis in HUVECs, whereas DAXX knockdown markedly repressed high Pi-induced cell apoptosis, indicating that DAXX mediated high Pi-induced endothelial cell apoptosis. High Pi treatment and DAXX overexpression induced the activation of extracellular regulated protein kinases (ERKs), while DAXX knockdown inhibited high Pi-induced ERKs activation. Finally, we demonstrated that DAXX overexpression induced HUVECs apoptosis in the presence of normal Pi, whereas additional treatment with U0126 (a specific ERK inhibitor) reversed that effect.

**Conclusion:**

Upregulated DAXX promoted high Pi-induced HUVECs apoptosis by activating ERK signaling and indicated that the DAXX/ERK signaling axis may be served as a potential target for CKD therapy.

## Introduction

Phosphorus, a necessary essential to all living things, is involved in a series of important life processes, including protein synthesis, signal transduction and energy metabolism (Kouloulias, Papaloucas & Papaloucas, 2019). Kidneys play a crucial role in the maintenance of phosphorus homeostasis (Suki & Moore, 2016). Phosphorus metabolism disorder is generally associated with chronic kidney disease (CKD) patients (Stremke & Hill Gallant, 2018). Long-term hyperphosphatemia results in secondary hyperparathyroidism (Lau, Obi & Kalantar-Zadeh, 2018), renal osteodystrophy (Rizk et al., 2017), and cardiovascular disease (CVD) (Molony & Stephens, 2011), and promotes the progression of nephropathy and increases the mortality rate of patients (Hannedouche, Fouque & Joly, 2018). The early detection of hyperphosphatemia and maintenance of normal plasma phosphorus levels is therefore of great significance.

High phosphate (Pi) can induce endothelial cell apoptosis and reactive oxygen species production, and reduces nitric oxide production, thus resulting in subsequent endothelial dysfunction (ED) (Di Marco et al., 2008; Shuto et al., 2009; Stevens et al., 2017; Tian et al., 2017). Mounting evidences has shown that hyperphosphatemia correlated with ED enhances cardiovascular morbidity and mortality in the patients with CKD (Disthabanchong, 2018; Hsu et al., 2015). A recent study identified a close correlation between extracellular regulated protein kinase (ERKs)/mitogen-activated protein kinase (MAPK) signaling and endothelial cell apoptosis (Qin et al., 2015). The suppression of ERK signaling attenuates endothelial dysfunction by enhancing the expression of BMP1 (bone morphogenetic protein 4) in high Pi-treated endothelial cells (Qin et al., 2015). Liu et al. (2013) reported that miR-155 reduces endothelial cell apoptosis by inhibiting Ang II-induced ERK1/2 activation.

Death domain-associated protein (DAXX), a multifunctional nucleoprotein, plays an important roles in the regulation of cell apoptosis, gene transcription, tumorigenesis and anti-viral infection (Dyer et al., 2017; Li et al., 2013; Li et al., 2017; Tang, Wan & Wu, 2015). However, the exact roles and underlying mechanisms of DAXX in cell apoptosis are not well understood. DAXX can bind to the death domain of FAS by activating the Jun amino-terminal kinase pathway to induce cell apoptosis (Villunger et al., 2000). DAXX also has an anti-apoptotic effect (Michaelson, 2000). Although DAXX functions in apoptotic and anti-apoptotic cell death, its precise role in this process remains unclear.

In the present study we investigated the role of DAXX in the regulating high Pi-induced human umbilical vein endothelial cell (HUVECs) apoptosis and identified its underlying mechanisms. It was found that high Pi upregulated DAXX expression in HUVECs. High Pi induced a significantly increased HUVECs apoptosis, whereas DAXX knockdown repressed that effect. High Pi induced the activation of extracellular regulated protein kinases, whereas DAXX knockdown inhibited high Pi-induced ERKs activation. We therefore, concluded that DAXX mediated high Pi-induced endothelial cell apoptosis by activating ERK signaling.

## Materials and Methods

### Animal experiments

Male SD rats (aged 6–8 weeks) were randomly divided into two groups: the CKD group (*n* = 5) and sham group (*n* = 5), All animals were obtained from the Shanghai Model Organisms Center, Inc. (Shanghai, China). All animal experiments were approved by the Ethic Committee of Shanghai Tenth People’s Hospital, School of Medicine, Tongji University (2016-33; Shanghai, China). In brief, rats were housed under constant environmental conditions with a 12-h light–dark cycle. Standard rat chow and water were provided ad libitum before and after surgery (Hsu et al., 2015). A two-step 5/6 nephrectomy was carried out to induce CKD as previously described (Hanifa et al., 2019; Uchiyama et al., 2016). Rats were anesthetized by peritoneal injection of pentobarbital (30–50 mg/kg) (Merck KGaA, Darmstadt, Germany) and right kidney was then exposed through a flank incision and ∼2/3 of the right kidney were removed. One week later, the left kidney was exposed and the upper and lower poles were resected through a flank incision. For sham rats, each flank of the kidney underwent sham surgery by incising the flanks, exposing the kidney and suturing each flank. Eight weeks after the second step of nephrectomy, rats were sacrificed and blood samples were obtained by cardiac puncture and urine was collected in individual metabolic cages.

### Biochemical measurements

The concentration of serum Pi and creatinine was assessed using an automated Olympus AU2700 chemistry analyzer (Tokyo, Japan). Urea in the urine was measured using Urea Assay Kit (MAK006, Merck KGaA) according to the manufacturer’s instructions.

### Cell culture and treatment

HUVECs were obtained from Lonza Group, Ltd. (Basel, Switzerland) and cultured with Endothelial Cell Growth Medium (ScienCell Research Laboratories Carlsbad, CA, USA) in a 37 °C incubator with 5% CO_2_. An appropriate amounts of Na2HPO4/NaH2PO4 buffer (1 M; pH7.4) was added into the cell culture medium to achieve different phosphorus concentrations (1.0 and 3.0 mM) with or without an inhibitor of Ca-Pi crystal formation, phosphonoformic acid (PFA, 1.0mM; Merck KGaA), as previously described (Di Marco et al., 2008; Peng et al., 2011; Zhou et al., 2016). Cells were treated with different Pi concentrations in the presence or absence of PFA for 24 h. ERK inhibitor U0126 (100 ng/mL, Thermo Fisher Scientific Inc, Waltham, MA, USA) was used to treat cells for 24 h when necessary, and DMSO was used as a vehicle control.

### Overexpression and Small Interfering RNAs (siRNAs)

The plasmids of pLEGFP-DAXX which encode the full-length DAXX sequence were constructed in our laboratory to overexpress DAXX in HUVECs. DAXX siRNA and negative control siRNA (NC-siRNA) were purchased from Shanghai GeneChem Co., Ltd. (Shanghai, China). The HUVECs in the logarithmic growth phase were inoculated into culture dishes before transfection, and the cells reached 60–80% confluence on the second day. Lipofectamine 3000 (Thermo Fisher Scientific, Inc.) was used to prepare the mixture of DAXX-siRNA, NC-siRNA, or pLEGFP-DAXX-lipofectamine 3000 according to the instructions. The mixture was then dropped evenly into each of the six well plates that already contained the cells and the culture medium. The transfection method was carried out in strict accordance with the instructions of the transfection kit. Pi or U0126 was used to treat the cells 48 h after transfection. The DAXX-siRNA sequence was 5′-GGAGTTGGATCTCTCAGAA-3′ and the NC-siRNA 5′-GUACCGCACGUCAUUCGUAUC-3′. The siRNA-treated cells were collected 48 h post-transfection for subsequent experiments.

### Flow cytometry

An annexin V-FITC/PI Apoptosis Detection Kit (Beyotime, Shanghai, China) was used to detect the apoptotic rate of cells according to the manufacturer’s instructions. In brief, HUVECs treated with 1.0 mM or 3.0 mM Pi and NC-siRNA or DAXX-siRNA for 24 h were washed with precooled culture medium twice, centrifuged at 2,000 rpm for 5 min to collect cells (3 ×10^5^), and then suspended with 500 µl binding buffer (culture medium with magnesium, calcium, 0.5% NaN3 and 1% FBS). Thereupon, Annexin V (5 µl) and PI (10 µl) were added to the cells and incubated for 10 min at room temperature in the dark. A flow cytometer (Attune NxT; Thermo Fisher Scientific, Inc) and ModFitLT software were used to detect and calculate the percentage of apoptotic cells.

### Quantitative PCR (qPCR)

Total RNA was extracted from HUVECs by Trizol reagent (Takara, Japan) and cDNA was synthesized using AMV reverse transcriptase (Merck Millipore, Billerica, MA, USA) according to the manufacturer’s instructions. qPCR was carried out by SYBR Green PCR Master Mix (Takara) on an Applied 7300 Real-Time PCR System (Thermo Fisher Scientific, Inc.). Relative mRNA levels were determined by normalization to the endogenous GAPDH mRNA. The sequence-specific primer pairs for DAXX was 5′-AGAAGCAAACAGGATCAGGG-3′ forward and 5′-GCTGGGCAGGGTACATATC-3′ reverse.

### Western blot analysis

The protein extracted from HUVECs lysate was detected using the BCA Protein Assay Kit (Thermo Fisher Scientific, Inc.), and an equal amount of protein was loaded and separated on 12% SDS-PAGE for each experiment. Following electrophoresis, the protein was transferred to PVDF membranes. The primary antibodies used included cleaved caspase-3 (1 µg/ml; ab2302), DAXX (1:5000, ab32140), ERK (1:1000, ab17942) and phospho-ERK(1:1000, ab201015) (all from Abcam, Cambridge, MA, USA). The membranes were incubated for 1 h with blocking buffer plus an horseradish peroxidase-conjugated goat anti-rabbit IgG H&L secondary antibody (1:10000; ab205718, Abcam). The band intensities were analyzed using Scion Image (WinB403).

### Immunohistochemistry

Frozen aorta tissues from a CKD rat model or sham rats embedded in optimal cutting temperature compound was cut into 5- µm-thick sections and fixed in 4% formaldehyde (Merck KGaA). Following washing 3 times with cold PBS, the sections were incubated with anti-Von Willebrand Factor (1:100, ab6994), and anti-DAXX (1:100, ab239806) primary antibodies (both from Abcam) overnight at 4 °C. Next, the sections were washed 3 times with PBS and incubated with Alexa Fluor 488 or Alexa Fluor 594-labeled secondary antibody for 1 h at room temperature in the dark. Three digital images were obtained from every aortic section using a confocal microscope and photographs were taken using Olympus confocal software (Olympus Corporation, Tokyo, Japan). The integrated optical density was assessed using Image-pro plus 4.5 software (Media Cybernetics, Inc., Rockville, MD, USA).

### Statistical analyses

Data are represented as the mean ± SEM of at least three independent experiments. The differences between two groups were compared using one–way ANOVA or Student’s *t*-test using GraphPad Prism statistical software (GraphPad Software, Inc., La Jolla, CA, USA). *P* < 0.05 was considered to indicate a statistically significant difference.

## Results

### Hyperphosphatemia was correlated with upregulated DAXX expression in endothelial cells

**Figure 1:**
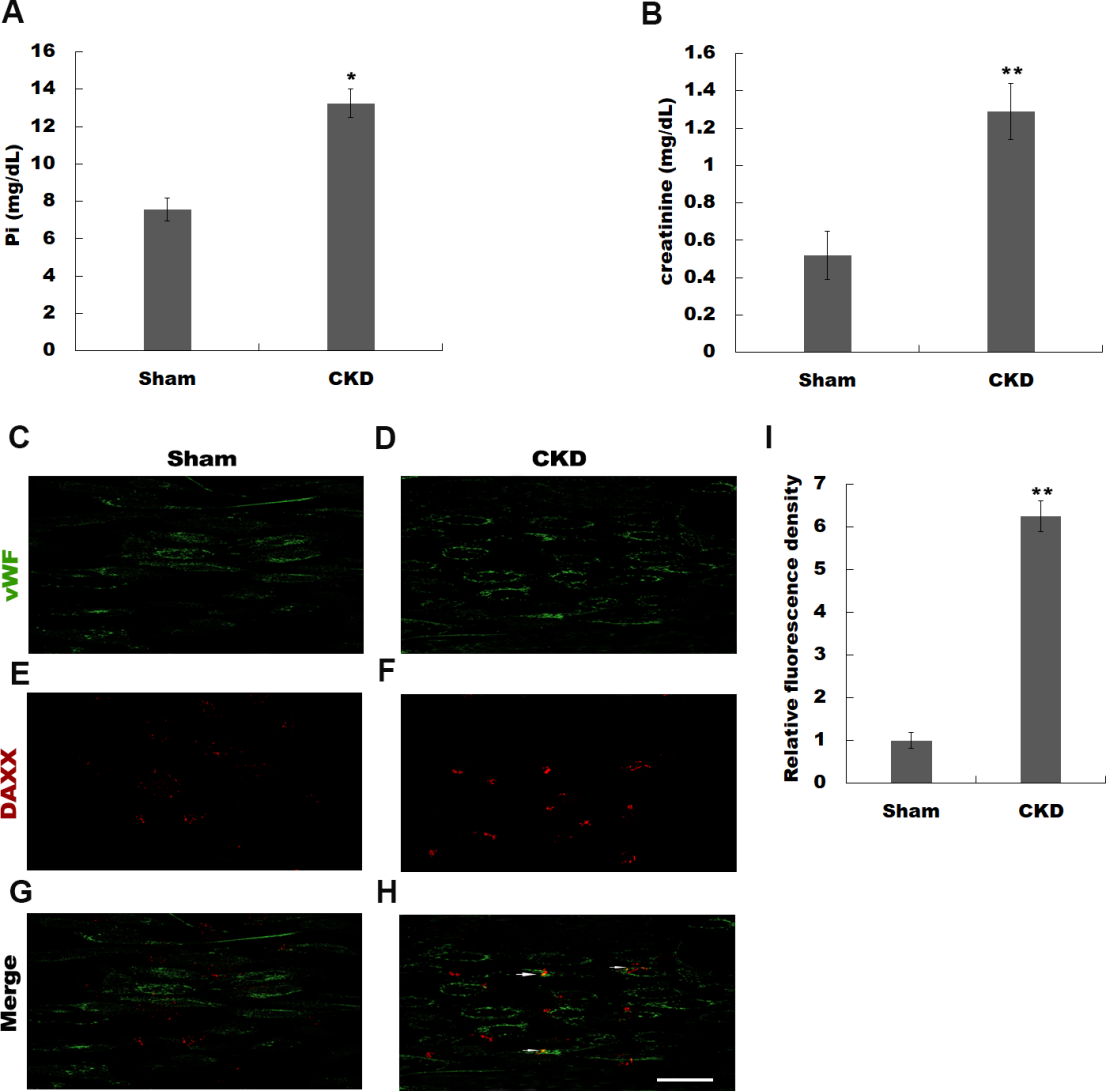
Hyperphosphatemia was correlated with upregulated DAXX expression in endothelial cells. The rat model of CKD was firstly established by 5/6 nephrectomy. Serum phosphate (Pi) (A) and creatinine (B) levels in CKD-rats and sham-operated rats were assessed by ELISA assay. The data are represented as mean ± SEM (n = 5). *p < 0.05, **p < 0.01. (C-I) Representative images and semiquantification of fluorescence intensity of immunohistofluorescence staining after double-stained with DAXX (red) and von Willebrand factor (vWF, green). Scar bar = 50 µm.

Previous studies have identified a number of differentially expressed genes using microarray analysis in high Pi-treated HUVECs (Qin et al., 2015). Qin et al. (2015) identified BMP4, ARPC1A, OR2D3, MYLK, GDF6, HPRT1, CDC45, ACSL1, TUBB2C, and DAXX as the top 10 differentially expressed genes in simulated hyperphosphatemia (Table S1). Given the important role of DAXX in regulating https://www.ncbi.nlm.nih.gov/pubmed/23771797 survival (Sakaue et al., 2017; Zeng et al., 2013), we attempted to investigate whether DAXX mediated high Pi-induced HUVECs apoptosis and identify its underlying mechanisms. The rat model of CKD was firstly established by 5/6 nephrectomy. Figures 1A and 1B show that CKD rats exhibited severe abnormalities in phosphorus metabolism and reveals a significant enhancement of serum creatinine, as compared with the sham-operated group. The urea level was increased in CKD rats as compared with Sham-operated rats (0.62 ± 0.11 vs 0.38 ± 0.09, *P* < 0.05). To assess whether DAXX is correlated with endothelial dysfunction in CKD rats, the DAXX expression in endothelial cells was examined using immunohistofluorescence. Figures 1C–1I shows that the staining intensity of DAXX protein in endothelial cells was obviously enhanced in CKD rats, compared with sham-operated group.

### High Pi resulted in a significant increase in DAXX expression

**Figure 2:**
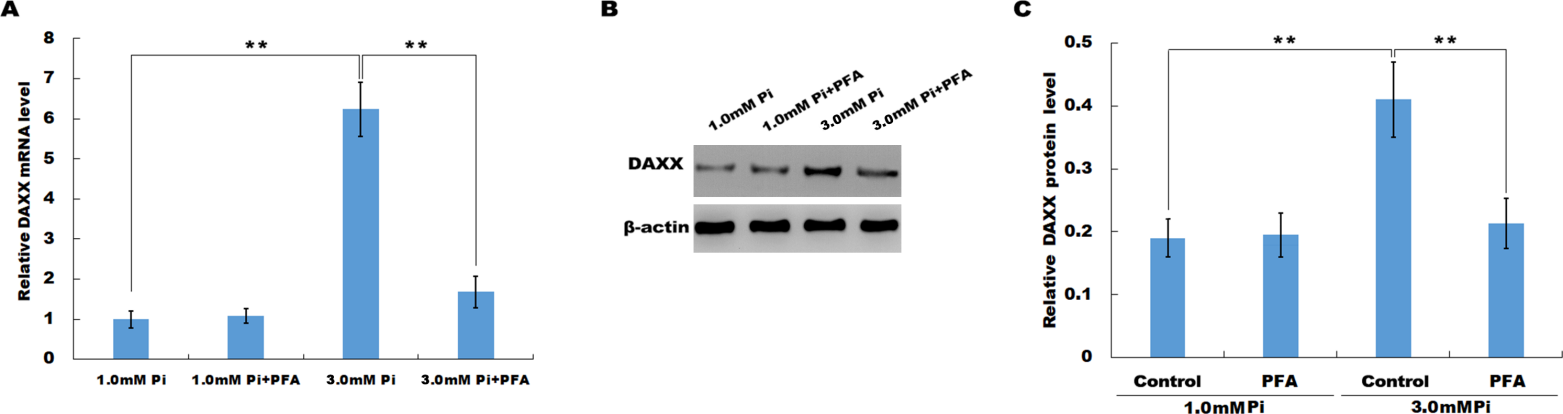
High Pi resulted in a significant increase of DAXX expression. (A) The mRNA level of DAXX in HUVEC cells was assessed using qPCR after treatment with different concentrations of Pi (1.0 mM and 3.0 mM) in the presence or absence of PFA (1mM). The 2^−ΔΔ*Ct*^ method was used to quantify relative DAXX level. Data are represented as the mean ± SEM of at least three independent experiments. (B and C) DAXX protein level in HUVEC cells was determined by western blot after treatment with different concentrations of Pi in the presence or absence of PFA. **p < 0.01.

In vitro, HUVECs were treated with normal or high Pi and the expression level of DAXX was then assessed using qPCR and western blot analysis. As shown in Fig. 2A, high Pi resulted in an increased mRNA expression of DAXX in HUVECs as compared with normal Pi. PFA (1 mM), an inhibitor of Ca-Pi crystal formation, reversed the high Pi-induced DAXX upregulation. Western blot analysis further verified that high Pi increased the protein level of DAXX in HUVECs as compared with normal Pi, and additional treatment of PFA reversed the high Pi-induced DAXX expression (Figs. 2B and 2C). These results demonstrated that high Pi resulted in a significant increase in DAXX expression.

### DAXX mediated high phosphate-induced endothelial cell apoptosis

**Figure 3:**
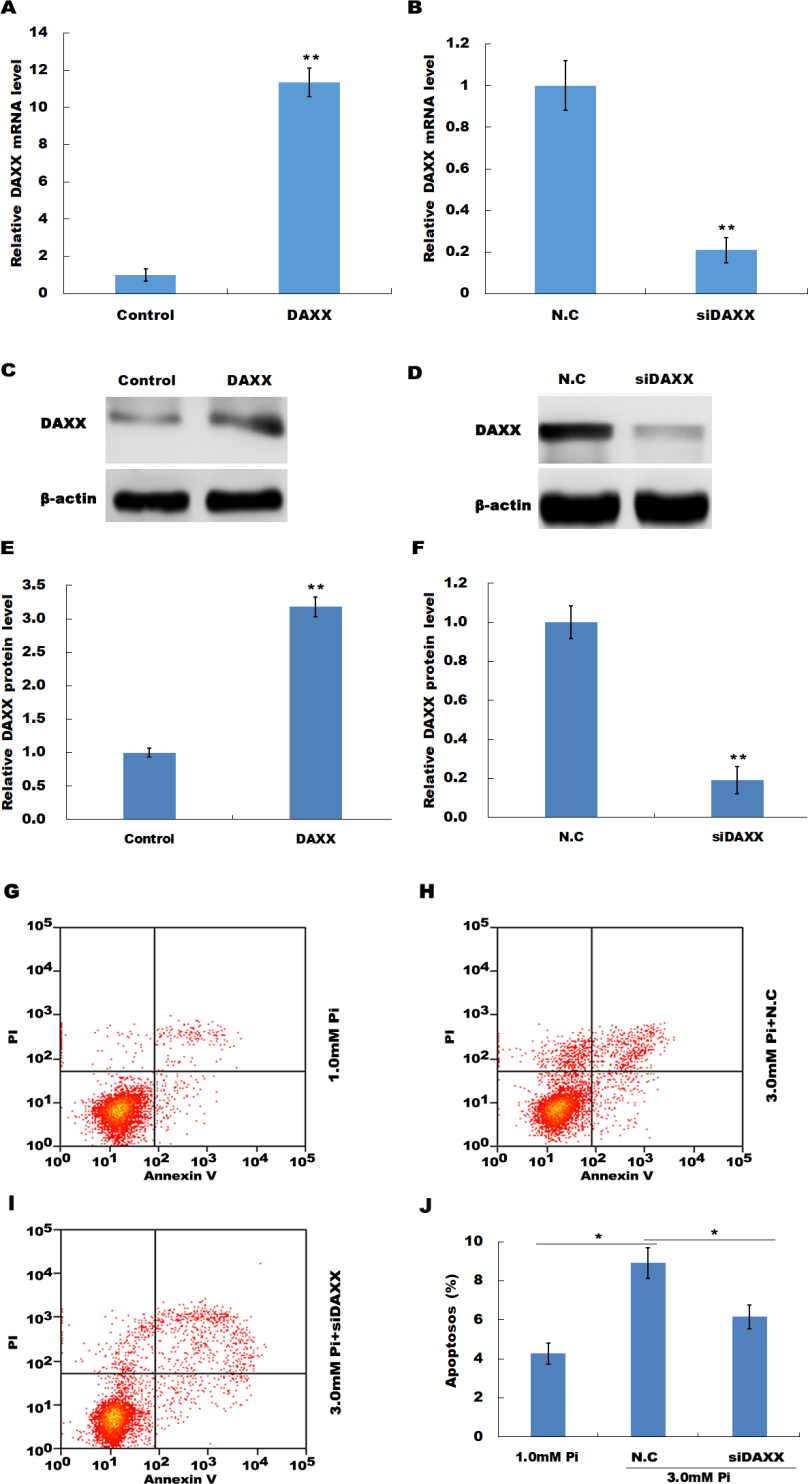
DAXX mediated high Pi-induced endothelial cell apoptosis. (A, C and E) qPCR and Western blot analysis for DAXX expression level in HUVECs cells after overexpression with DAXX. (B, D and F) qPCR and western blot analysis for DAXX expression level in HUVECs cells after DAXX inhibition. (G–I) Cell apoptosis was assessed using flow cytometric analysis in normal Pi environment, and high Pi in the presence or absence of siDAXX. (J) The apoptosis rate was quantified by FACS software, and data are represented as the mean ± SEM of at least three independent experiments. *p < 0.05. **p < 0.01.

The frequent increase of DAXX in simulated hyperphosphatemia implied that DAXX may plays a regulatory role in disease progression. Therefore, the biological effect of DAXX in regulating HUVECs apoptosis was then examined. The results from qPCR and western blot analysis showed that DAXX level was significantly upregulated in HUVECs following DAXX overexpression (Figs. 3A, 3C and 3E). As expected, DAXX was markedly downregulated in HUVECs treated with DAXX-specific siRNAs (Figs. 3B, 3D and 3F). Functionally, the results from flow cytometry showed that high Pi resulted in a significant increase of HUVECs apoptosis, whereas DAXX knockdown repressed high Pi-induced cell apoptosis (Figs. 3G–3J).

**Figure 4:**
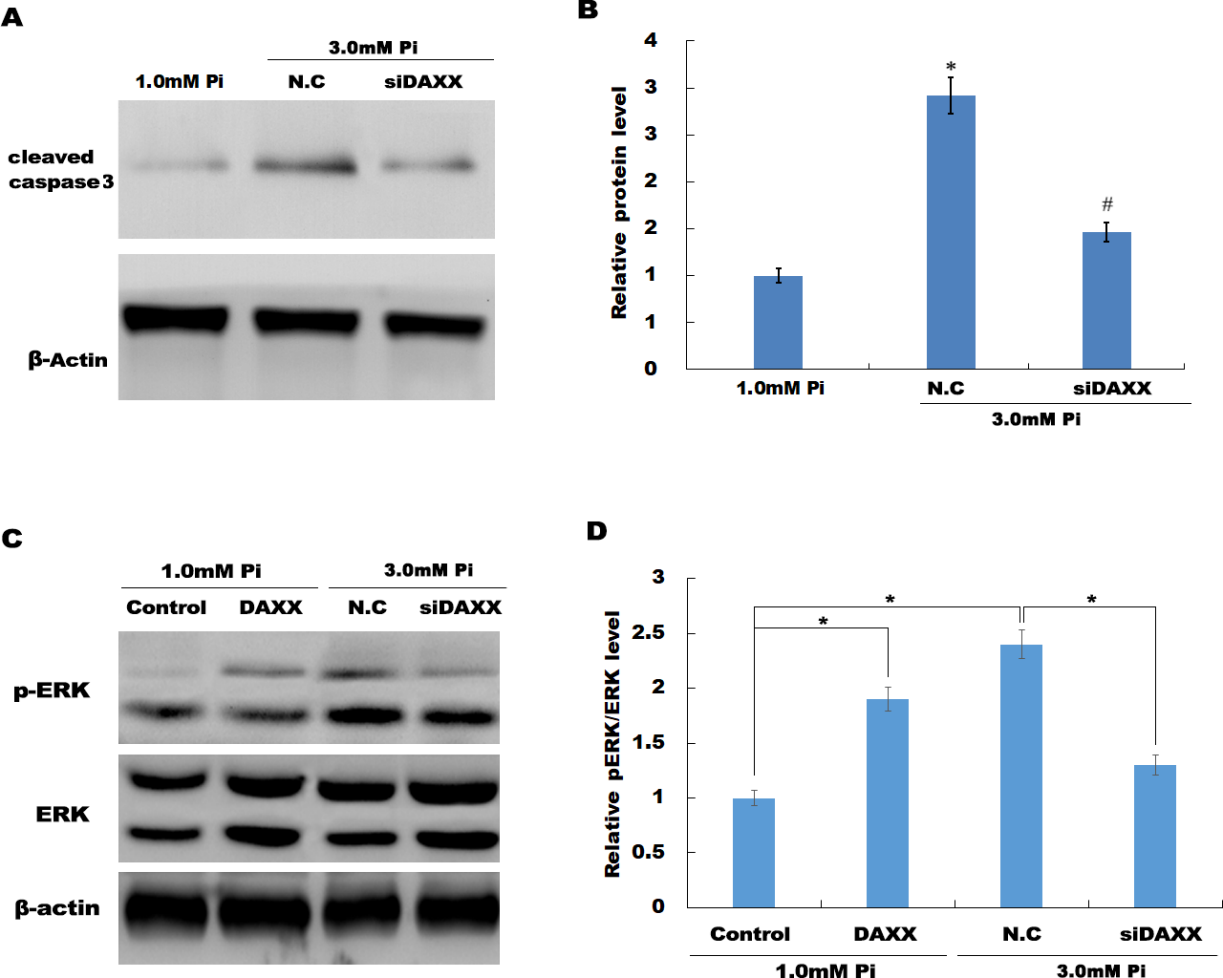
DAXX activated ERK signaling in HUVECs. (A and B) HUVECs were treated with normal Pi and high Pi in the presence or absence of siDAXX, and then the protein level of cleaved caspase-3 was assessed using western blot analysis. (C and D) The phosphorylation of ERK was detected in HUVEC cells treated with normal Pi or high Pi for 1h in the presence of DAXX overexpression or inhibition. *p < 0.05 compared to control group; #*p* < 0.05 compared to the NC-siRNA group.

In HUVECs treated with high Pi, a significant activation of cleaved caspase-3 was observed as compared with that in HUVECs treated with normal Pi, whereas knockdown of DAXX markedly inhibited high Pi-induced cleaved caspase-3 activation, further indicating the role of DAXX in regulating high Pi-induced HUVECs apoptosis (Figs. 4A and 4B).

### DAXX mediated high Pi-induced HUVECs apoptosis through activating ERK signaling

**Figure 5:**
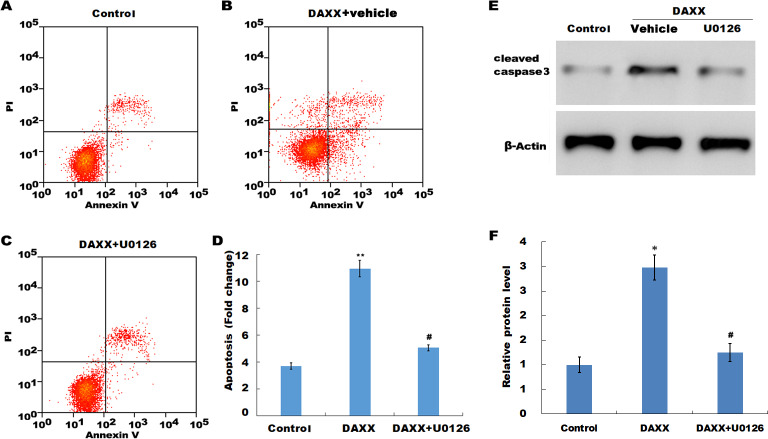
DAXX mediated high Pi-induced HUVECs apoptosis through activating ERK signaling. (A–C) HUVECs were overexpressed with DAXX in the presence or absence of U0126 (100 ng/mL), and then cell apoptosis was assessed using flow cytometric analysis. (D)The apoptosis rate was quantified by FACS software, and data are represented as the mean ± SEM of at least three independent experiments. (E and F) HUVECs were overexpressed with DAXX in the presence or absence of U0126 (100 ng/mL), and then the protein level of cleaved caspase-3 was assessed using western blot analysis. *p < 0.05 compared to control group; **p < 0.01 compared to control group; #*p* < 0.05 compared to the overexpression DAXX group.

Previous studies have reported that MAPK signaling is the most prominently aberrant pathway in high Pi-treated HUVECs, and ERK activation contributes to endothelial cell apoptosis. The present study investigated the association between DAXX and ERK activation. The western blot analysis results showed that DAXX overexpression upregulated the phosphorylated ERK (p-ERK) activity in the presence of normal Pi (Figs. 4C and 4D). Furthermore, high Pi resulted in a significant increase in p-ERK activity in HUVECs as compared with normal Pi, whereas DAXX knockdown markedly repressed the high Pi-induced activation of ERK signaling (Figs. 4C and 4D), demonstrating the regulatory role of DAXX in the activation of ERK signaling. Functional studies further showed that DAXX overexpression in HUVECs promoted cell apoptosis in the presence of normal Pi, whereas additional treatment with U0126, a specific inhibitor of ERK activation, for 24 h inhibited that effect (Figs. 5A–5D). Western blot analysis also demonstrated that DAXX overexpression in HUVECs resulted in a significant activation of cleaved caspase-3 in the presence of normal Pi, whereas additional treatment with U0126 markedly inhibited, the DAXX-induced activation of cleaved caspase-3 (Figs. 5E and 5F). These results confirmed that DAXX mediated high Pi-induced endothelial cell apoptosis by activating ERK signaling.

## Discussions

Pi is an indispensable element in most biologic processes such as DNA/RNA synthesis, signal transduction and energetic metabolism (Shobeiri, Adams & Holden, 2014; Tan et al., 2016). However, the aberrant accumulation of Pi is detrimental. Pi accumulation possibly proceed long since serum Pi raises above the normal range (Disthabanchong, 2018). Long-term Pi load eventually leads to extensive vascular calcification in end-stage kidney disease (Disthabanchong, 2018). Enhanced Pi can accelerate VSMC (vascular smooth muscle cell) apoptosis, and facilitate phenotypic switching into osteogenic cells which exerts a critical effect on vascular calcification (Hsu et al., 2015; Moe & Chen, 2004). Emerging studies have showed that endothelial cells produce more ROS and reduces the release of nitric oxide after treatment with high Pi, and thus results in cell apoptosis (Peng et al., 2011). Acute serum Pi enhancement after postprandial reduces endothelial cell-driven vasodilation, also indicating the close correlation between high Pi-induced ED and cardiovascular mortality risk (Shuto et al., 2009). However, the role of high Pi in endothelial cell damage remains poorly understood. The present results verified the important role of DAXX in regulating high Pi-induced HUVECs apoptosis and may provide a novel opportunity to treat high Pi-induced endothelial cell apoptosis.

DAXX can both promote and suppress apoptosis (Tang, Wan & Wu, 2015), and the specific mechanism of DAXX remains unclear. DAXX largely localizes to the nucleus of un-stimulated cells and exerts an anti-apoptotic effect in the nucleus (Tang, Wan & Wu, 2015). Dionne et al. (2013) demonstrated that DAXX has an anti-apoptotic effect on the nucleus and promotes cell apoptosis in reovirus-infected cells cytoplasm. DAXX in the nucleus could repress p53 phosphorylation and affect cell cycle progression (Kumar et al., 2013). DAXX was transferred from the nucleus to the cytoplasm and its functions were changed when DAXX mutated in an NLS domain (Yeung et al., 2008). The overexpression of DAXX may cause it to aggregate in the cytoplasm and bind to Fas and other death receptors to promote apoptosis (Yang et al., 1997). Emerging studies reported the pro-apoptotic role of DAXX in HUVECs following treatment with a chemotherapeutic drug or oxidative stress (Salomoni & Khelifi, 2006; Zeng et al., 2013). Zeng et al. (2013) demonstrated that DAXX is upregulated in high glucose-treated HUVEC cells following miR-21 knockdown and contributes to HUVEDs apoptosis. However, the function of DAXX in regulating high Pi-induced endothelial cell apoptosis and its underlying mechanism remains unknown.

In the present study, we first assessed the aberrant expression of DAXX in HUVECs following high Pi treatment. It was found that high Pi resulted in the increase of DAXX expression in HUVECs as compared with normal Pi, and PFA abrogated high Pi-induced DAXX upregulation. Functionally, the results from flow cytometry showed that high Pi resulted in a significant increase of HUVECs apoptosis, whereas DAXX knockdown markedly repressed high Pi-induced cell apoptosis, indicating that DAXX mediated HUVECs apoptosis in the presence of high Pi.

Previous studies have demonstrated that multiple pathways of MAPKs are involved in the proliferation, differentiation and apoptosis of endothelial cells under different conditions, which leads to the disruption of apoptosis and regeneration balance of endothelial cells as well as the occurrence of cardiovascular events (Liu et al., 2018; Niaudet et al., 2017). However, the specific molecular mechanism of high Pi-induced endothelial cells injury remains unclear, and the role of MAPK signaling in endothelial cells of patients with hyperphosphatemia is not well understood. Given the important role of ERK/MAPK signaling in regulating endothelial cells apoptosis, the present study investigated the association between DAXX and ERK activation. Current data showed that DAXX overexpression resulted in p-ERK activity in the presence of normal Pi. Furthermore, DAXX knockdown markedly repressed the high Pi-induced activation of ERK signaling. Functionally, DAXX overexpression in HUVECs promoted cell apoptosis in the presence of normal Pi, whereas additional treatment with U0126 inhibited that effect.

## Conclusion

In this study no effect of DAXX on the regulation of p38 and JUN signaling was detected. Whether they are involved in the high Pi-induced endothelial cell apoptosis requires further study. These data demonstrated that DAXX mediated high Pi-induced endothelial cell apoptosis by activating ERK signaling, indicating that the DAXX/ERK signaling axis may be served as a potential target for hyperphosphatemia.

##  Supplemental Information

10.7717/peerj.9203/supp-1 Data S1 Raw Data

10.7717/peerj.9203/supp-2 Table S1 Top ten differentially expressed genes in simulated hyperphosphatemia

## Additional Information and Declarations

### Data Availability

The following information was supplied regarding data availability: The raw measurements are available in the Supplemental Files.
