# Supplementary figures and images for: DAXX mediates high phosphate-induced endothelial cell apoptosis in vitro through activating ERK signaling
Source: PeerJ. 2020 Jun 19;8:e9203. doi: 10.7717/peerj.9203 (PMC7307556; doi:10.7717/peerj.9203)

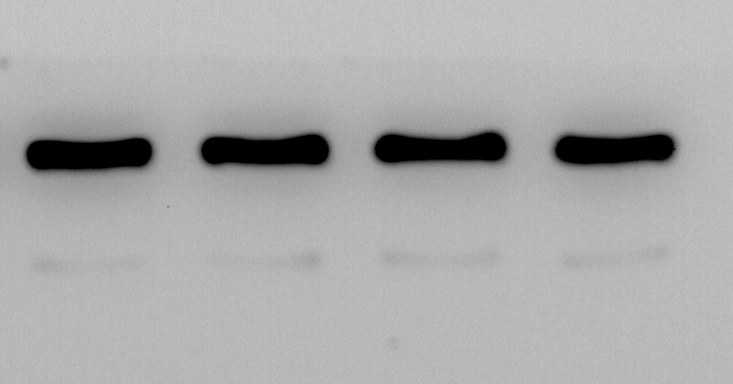

Supplement: Data S1 [file peerj-08-9203-s001.zip › Raw Data/WB/Fig2/Actin.jpg]

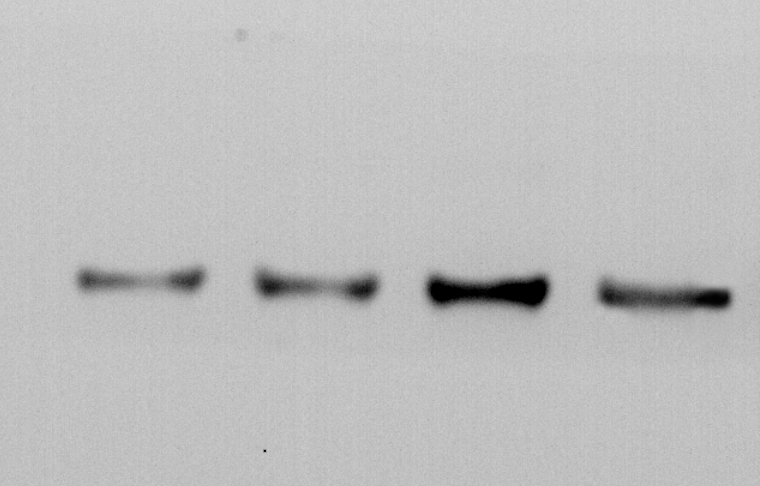

Supplement: Data S1 [file peerj-08-9203-s001.zip › Raw Data/WB/Fig2/DAXX.jpg]

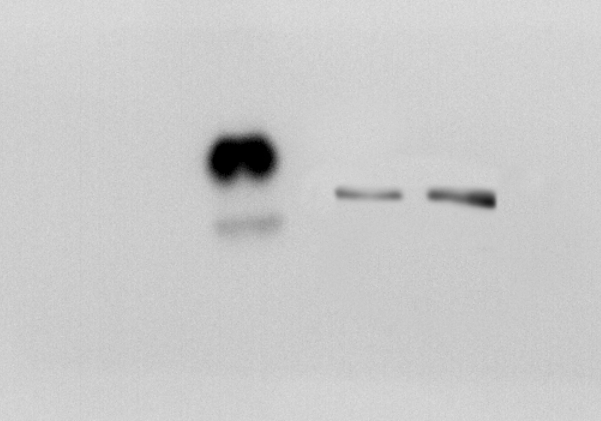

Supplement: Data S1 [file peerj-08-9203-s001.zip › Raw Data/WB/Fig3/DAXX-1.tif]

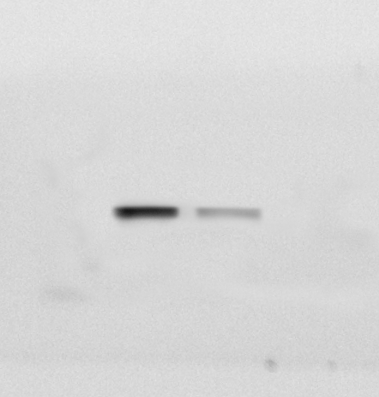

Supplement: Data S1 [file peerj-08-9203-s001.zip › Raw Data/WB/Fig3/DAXX-2.tif]

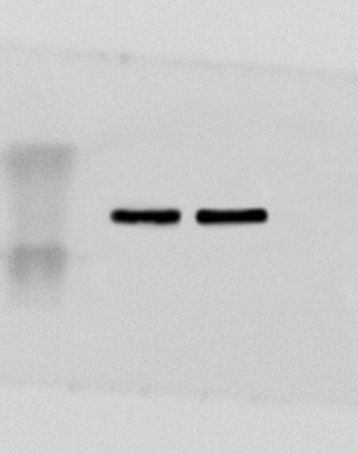

Supplement: Data S1 [file peerj-08-9203-s001.zip › Raw Data/WB/Fig3/actin-1.tif]

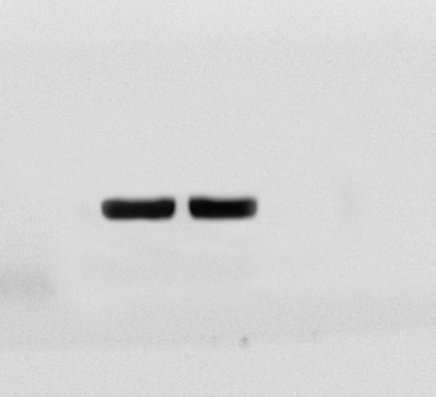

Supplement: Data S1 [file peerj-08-9203-s001.zip › Raw Data/WB/Fig3/actin-2.tif]

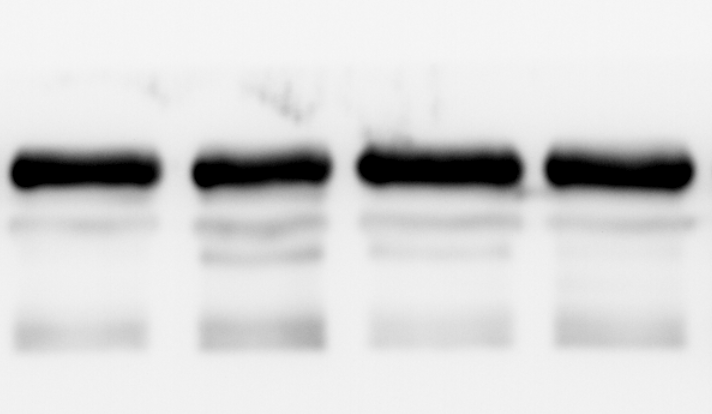

Supplement: Data S1 [file peerj-08-9203-s001.zip › Raw Data/WB/Fig4/ACTIN-2.tif]

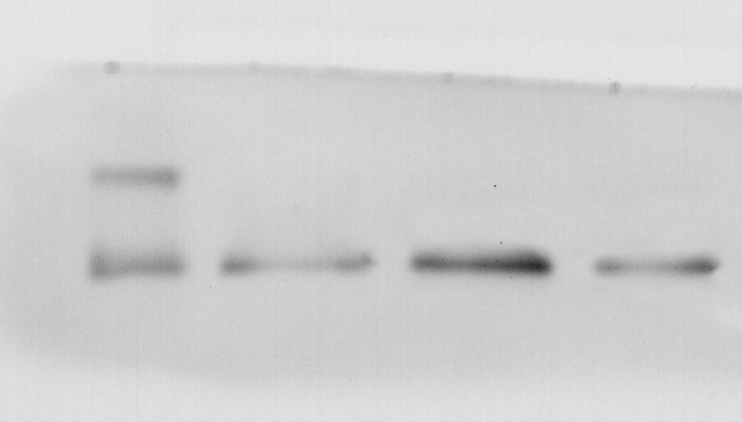

Supplement: Data S1 [file peerj-08-9203-s001.zip › Raw Data/WB/Fig4/C-caspase3.tif]

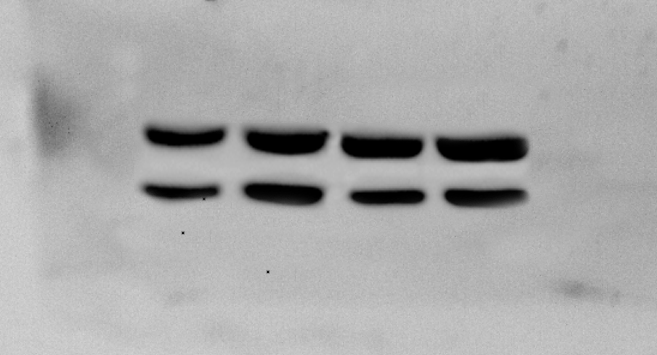

Supplement: Data S1 [file peerj-08-9203-s001.zip › Raw Data/WB/Fig4/ERK.tif]

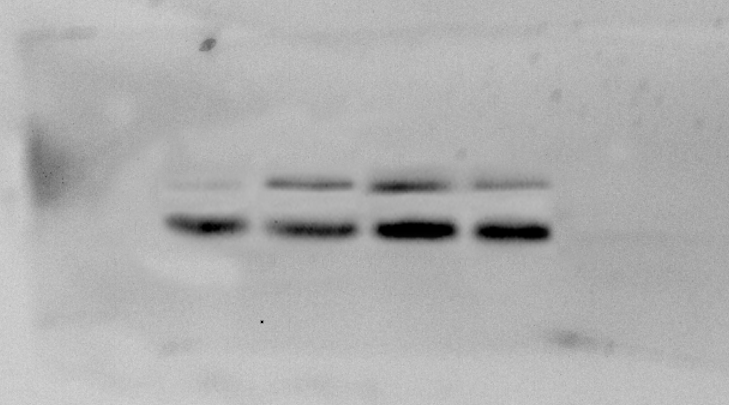

Supplement: Data S1 [file peerj-08-9203-s001.zip › Raw Data/WB/Fig4/PERK.tif]

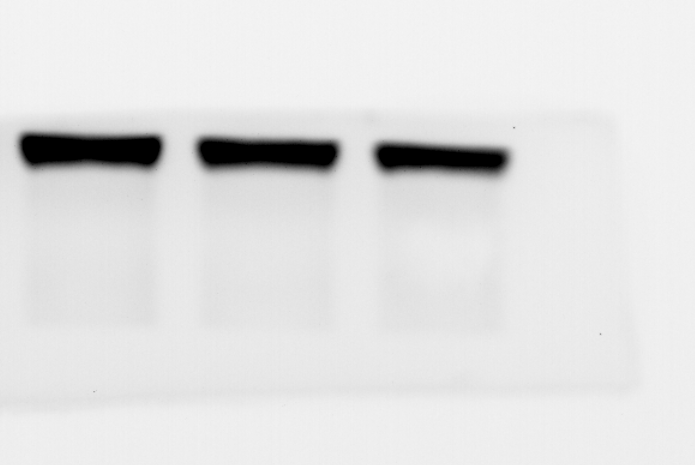

Supplement: Data S1 [file peerj-08-9203-s001.zip › Raw Data/WB/Fig4/actin-1.tif]

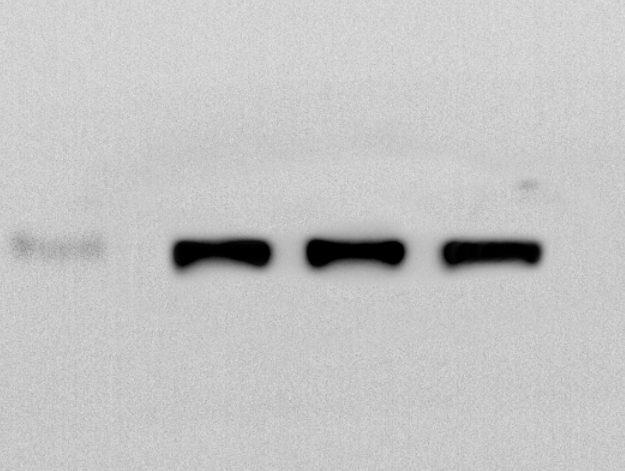

Supplement: Data S1 [file peerj-08-9203-s001.zip › Raw Data/WB/Fig5/actin.tif]

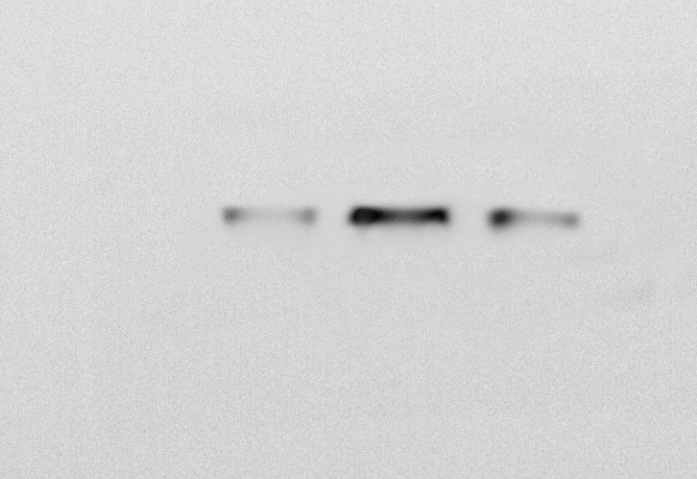

Supplement: Data S1 [file peerj-08-9203-s001.zip › Raw Data/WB/Fig5/c-casp3.tif]
