# Supplementary material for: DAXX mediates high phosphate-induced endothelial cell apoptosis in vitro through activating ERK signaling
Source: PeerJ. 2020 Jun 19;8:e9203. doi: 10.7717/peerj.9203 (PMC7307556; doi:10.7717/peerj.9203)
Supplement: Table S1 [file peerj-08-9203-s002.docx]

**Supporting Table S1.** **Top ten differentially expressed genes in simulated hyperphosphatemia.**

| Gene | Description | Total degree | k-core |
| --- | --- | --- | --- |
| BMP4 | Bone morphogenetic protein 4 | 15 | 10 |
| ARPC1A | Actin related protein 2/3 complex, subunit 1A | 15 | 10 |
| OR2D3 | Olfactory receptor, family 2, subfamily D, member 3 | 15 | 10 |
| MYLK | Myosin light chain kinase | 15 | 10 |
| GDF6 | Growth differentiation factor 6 | 15 | 10 |
| HPRT1 | Hypoxanthine phosphoribosyltransferase 1 | 15 | 10 |
| CDC45 | Cell division cycle 45 homolog (S. cerevisiae) | 14 | 10 |
| ACSL1 | Acyl-CoA synthetase long-chain family member 1 | 14 | 10 |
| TUBB2C | Tubulin, beta 2C | 20 | 9 |
| DAXX | Death-domain associated protein | 19 | 9 |
